# The Effects of Sea and Road Transport on Physiological and Electroencephalographic Responses in Brahman Crossbred Heifers

**DOI:** 10.3390/ani9050199

**Published:** 2019-04-27

**Authors:** Idrus Zulkifli, Ahmed A. Abubakar, Awis Q. Sazili, Yong M. Goh, Jurhamid C. Imlan, Ubedullah Kaka, Azad B. Sabow, Elmutaz A. Awad, Azalea H. Othman, Razlina Raghazali, Clive J.C. Phillips, Hassan N. Quaza Nizamuddin, Helen Mitin

**Affiliations:** 1 Institute of Tropical Agriculture and Food Security, Universiti Putra Malaysia, UPM Serdang 43400, Selangor, Malaysia; ahmadsadeeq7@gmail.com (A.A.A.); awis@upm.edu.my (A.Q.S.); ymgoh@upm.edu.my (Y.M.G.); jurhamidimlan@yahoo.com.ph (J.C.I.); dr_ubedkaka@upm.edu.my (U.K.); elmutazatta@upm.edu.my (E.A.A.); 2 Department of Animal Science, Faculty of Agriculture, Universiti Putra Malaysia, UPM Serdang 43400, Selangor, Malaysia; azad1979sabow@yahoo.com; 3 Halal Products Research Institute, Universiti Putra Malaysia, UPM Serdang 43400, Selangor, Malaysia; 4 Department of Preclinical Sciences, Faculty of Veterinary Medicine, Universiti Putra Malaysia, UPM Serdang 43400, Selangor, Malaysia; razlina81@gmail.com; 5 Department of Animal Science, College of Agriculture, University of Southern Mindanao, Kabacan 9407, North Cotabato, Philippines; 6 Department of Animal Resource, College of Agriculture, Salahaddin University-Erbil, Kurdistan Region 44002, Iraq; 7 Department of Poultry Production, Faculty of Animal Production, University of Khartoum, Khartoum 13314, Sudan; 8 Department of Veterinary Pathology and Microbiology, Faculty of Veterinary Medicine, Universiti Putra Malaysia, UPM Serdang 43400, Selangor, Malaysia; azalea@upm.edu.my; 9 Building 8143, Centre of Animal Welfare and Ethics (CAWE), School of Veterinary Science, University of Queensland, Gatton, Queensland 4343, Australia; c.phillips@uq.edu.au; 10 Department of Veterinary Services, Wisma Tani, Blok Podium, Lot 4G1, No. 28, Persiaran Perdana, Presint 4, Federal Government Administrative Centre, Putrajaya 62630, Malaysia; quaza@dvs.gov.my (H.N.Q.N.); helenmitin21@gmail.com (H.M.)

**Keywords:** Brahman crossbred cattle, sea and road transport, welfare, cortisol, acute phase proteins, haematological indicators, blood biochemical, electroencephalographic response

## Abstract

**Simple Summary:**

This study investigated the effect of sea and road transport on the physiological response of cattle. The animals were transported by sea (14 d) from Darwin Port, Australia, to Pasir Gudang Port, Johor, Malaysia. Thereafter, the animals were road transported (330 km) from Pasir Gudang Port to Universiti Putra Malaysia (UPM). From the results of the various blood stress indicators and brain activity of the animals, both sea and road journeys were found to be stressful to the animals. However, the animals recovered from the stressful transport journeys following 4 and 7 days post-transport.

**Abstract:**

The objective of the current study was to evaluate the effects of sea and road transport on the acute phase proteins (APP), cortisol, metabolic, haematological and electroencephalographic (EEG) responses of Brahman crossbred heifers. Sixty Brahman crossbred heifers were subjected to 14 d of transportation by sea from Darwin Port, Australia, to Pasir Gudang Port, Johor, Malaysia, and 330 km of road transportation. Results revealed that the intensity of response for most blood biochemical parameters increased significantly and were different from the baseline values taken while the animals were in Darwin Port, Australia. Haematological results obtained also revealed a significant increase and were different from the baseline values. Cortisol and APP (bovine alpha 1-acid glycoprotein and serum amyloid-A) values increased significantly and were different from the baseline values. Haematological parameters, APP, cortisol and EEG data (alpha, beta, delta and theta waves, total power and median frequency) decreased significantly following 4 and 7 days post-transport, suggesting a recovery of the animals from the stressfulness of transport. In conclusion, the current results revealed that the concentrations of biochemical and haematological parameters, cortisol, APP and EEG data were affected by both sea and road transport as evidenced by the significant changes recorded from the parameters above.

## 1. Introduction

There is increasing transport of livestock through long distances as demand for meat products increases, particularly in Asia; freight opportunities grow and trade barriers are reduced [1]. Most of this transport is by road and sea. During transport, the animals are subjected to several physical and psychological stressors, such as thermal extremes, handling by humans, floor movement, restriction of movement and feed, noise, crowding and social disruption [2,3]. The processes of loading and unloading animals into and out of the transport vehicles are a particularly high-risk period [4,5]. The adverse impacts on animal welfare caused during transportation include thermal and physical discomfort, stress, muscular damage and dehydration, and behavioral and movement limitations [6]. Although there is a considerable amount of documented work on road transport in farm animals, most studies have been conducted in temperate regions. In tropical and equatorial regions, heat stress is a particularly big risk. As a result, it is necessary to consider changes to the existing practices of animal transportation in terms of animal welfare when the animals are transported in hot and humid tropical environments. Earlier work on the welfare of road-transported farm animals in Malaysia was on poultry (1 to 6 h of transport) [7,8,9,10,11], goats (1 to 3 h of transport) [12,13] and rabbits (1 to 3 h of transport) [14], all of which are prone to high mortality during long distance transport. As a result, long-distance transport of these animals is rare. Increasingly common, however, is the long-distance transport of cattle from Australia to South Asian countries, such as Vietnam and Malaysia, and little research has been done to investigate the physiological responses of the animals.

In 2017, Malaysia imported 25,585 live cattle from Australia, of which 23,542 were beef cattle [15], in a journey that necessarily involves both sea and road transport. Before embarking on the ship, usually in Darwin, cattle are mustered from remote farms by airplane, helicopter and vehicles into yards, from which they are transported by road to a holding yard before loading at a suitable date. The ship voyage from Australia to Malaysia is about two to three weeks. The primary challenges experienced by cattle transported by sea from Australia to Asia include trauma, respiratory disease, conjunctivitis and heat stress [16]. The current study was carried out to analyze the impacts of sea and road transport on the welfare of Brahman cross cattle transported from Australia to Malaysia. A particular focus of this study was the ability of the animals to recover after sea and road transport. For this purpose, as well as standard physiological and haematological indicators [17,18,19,20,21], we also measured electroencephalographic (EEG) responses to the road transportation component. To the best of our knowledge, this is the first documented work on EEG and non-pain stimulus in cattle. Work in human beings showed EEG responses to non-painful warn and cold stimuli [22]. Tops et al. [23], and McAllister-Williams et al. [24] demonstrated a relationship between exogenous cortisol administration and EEG response in human beings. Hence, it appears non-painful stressful stimuli may induce circulating levels of cortisol and subsequently elicit EEG reaction in humans. Physiological indicators and EEG response give a comprehensive assessment of the impact of the transport on cattle. Our hypothesis was that both sea and road transport will elicit physiological and EEG responses and animals will recover within seven days post-transport.

## 2. Materials and Methods

The experimental protocol was approved by the institutional animal care and use committee (IACUC) of the Universiti Putra Malaysia (UPM/IACUC/R028/2016).

### 2.1. Animals, Transport and Treatment

Sixty Brahman crossbred heifers of about 18 months of age (mean live weight 240 ± 50 kg) were sourced from Katherine, a town located in the Northern Territory of Australia. A total of 60 animals were imported from Australia to allow random blood sampling at each point and for a separate experiment. Brahman crossbred is the most common imported beef cattle breed in Malaysia because of its ability to withstand the hot environment. It normally takes two days for mustering, holding in yards and delivery to the pre-assembly depot. The animals were road transported for 306 km to the Berrimah Quarantine Inspection Centre on 29 February, 2016. Upon arrival, the animals were unloaded and held in pens with ad libitum feed and water. The mean dry bulb temperatures in the quarantine facility were 28–31 °C during the day and 22–24 °C at night; relative humidity was 80%. On 6 March, 2016, 24 heifers were randomly selected and blood samples (≈10 mL) were taken by coccygeal venepuncture into blood collection tubes. Serum samples were separated by centrifugation at 1600 × *g* at 8 °C for 15 min, and subsequently stored at −80 °C until they were air freighted to Universiti Putra Malaysia (UPM). On 11 March, 2016, the animals were road transported 12 km to Darwin and loaded into a vessel. This livestock carrier, which was built in 2006 (Xixiakou Shipbuilding, Rongcheng, China) and operated out of Darwin by Karumba Livestock Holding, had a gross tonnage of 2964 t, dimensions 81 × 13.6 m, with two naturally ventilated decks and two artificially ventilated decks at or below sea level. Details of the housing condition in the vessel are not available.

The sea voyage to Pasir Gudang Port in Johor, Malaysia, covered approximately 3362 km at a speed of 10 knots, over a duration of 14 days. On the route to Malaysia, the vessel transited at Brunei for 48 h. Upon arrival (07:00 h) at Pasir Gudang Port, Johor, the animals were inspected by quarantine officers and remained in the vessel for 12 h before being unloaded. Feed and water were available until disembarkation. At the port, dry bulb temperatures on that day were maximum 35.3 °C and 23.4 °C minimum, with a relative humidity of 81.6% (Malaysian Department of Meteorology). Prior to disembarkation from the vessel, 24 animals were randomly selected and blood samples taken by coccygeal venepuncture into blood collection tubes for serum and plasma (with heparin as an anticoagulant). Samples collected were stored temporarily in crushed ice for about an hour and later centrifuged at 1000 × *g* for 10 min. The plasma clots as well as the serum were separated in replicates of 1.5 mL aliquots and stored at −80 °C until analysis.

Following blood sampling, the animals disembarked and were loaded onto trucks at 22:00 h, and then transported for 7.00 h (330 km) by road to the Animal Research Centre of the Institute of Tropical Agriculture and Food Security, Universiti Putra Malaysia (UPM), Serdang, Selangor. The animals were unloaded from the ship and loaded onto the trucks using non-slip loading ramps with stair-steps built from wood. The angle of the ramp was about 20°. Similar ramps were used to unload the animals from the truck at UPM. The transportation of animals took place in two different trucks, each with a carrying capacity of 30 cattle, at a space allowance of 0.96 m^2^ per animal. The trucks travelled the highway at an average speed of 80 km/h. Upon arrival at UPM (24.5–25.5 °C with relative humidity of 84.4%), the animals were unloaded and 24 animals were randomly selected for blood sample collection by the same technique as the initial sampling.

### 2.2. Housing and Management

On arrival at the Animal Research Centre, the animals were randomly assigned to groups of 15 to four separate pens in a naturally ventilated house. The floor area of each pen was 52.5 m^2^ and it had a concrete floor and aluminum roofing. All the 60 animals were subjected to a similar travel chain from Australia to UPM and, thus, it is likely that the animals in each pen were familiar with each other. The animals had access to commercial beef cattle feed (97.5% palm kernel cake; 1.5% limestone; 1.0% mineral premix), grass pellets, rice straw, and drinking water. The feeding space allowed was 120 cm per animal. Supplementary lighting provided ensured continuous lighting. Over the seven days post-road transport, the mean maximum and minimum daily dry bulb temperatures and relative humidity were 35.3, 23.4 °C and 81.6%, respectively (Malaysian Department of Meteorology). Blood sampling procedures detailed earlier were repeated at 4 and 7 days post-road transport. Twenty-four randomly selected animals were used for each occasion of blood sampling. The sampling intervals were chosen based on earlier work [21], which demonstrated that physiological and haematological indicators returned to pre-transport values within 48 h of recovery while acute phase proteins required 7 days to return to control values in cattle subjected to long-distance transportation.

### 2.3. Physiological and Haematological Indicators

Serum concentrations of cortisol (CORT) (My-Biosource, San Diego, CA, USA), alpha-1 acid glycoprotein (AGP) (Immunology Consultant’s Laboratory Inc., Portland, OR, USA), amyloid-A (AA) (Tridelta Development Ltd, County Kildare, Ireland) were determined using the appropriate Enzyme-Linked Immuno Sorbent Assay (ELISA) kits. An automatic analyzer (Hitachi 902, Tokyo, Japan) was used to determine serum levels of alkaline phosphatase (ALP), creatine kinase (CK), aspartate aminotransferase (AST), lactate dehydrogenase (LDH) and alanine aminotransferase (ALT). All the reagents used were obtained from Roche (Roche Diagnostics, Basel, Switzerland).

On a microscope glass slide (25 mm × 75 mm and thickness of approximately 1 mm), a thin layer of blood was smeared and stained with methylene blue. The glass slide was then observed and assessed under an electronic microscope, and with the help of an automatic haematology analyzer (CELL DYN^®^ 3700, Abbott, Lake County, IL, USA), the constituent white blood cells (WBC), red blood cells (RBC), packed cell volume (PCV), haematocrit, haemoglobin, platelets, and neutrophils and lymphocytes counts were ascertained using the Veterinary Package software. All reagents used were from Abbott (Abbott, Lake County, IL, USA. The neutrophil:lymphocyte (N:L) ratio was calculated.

### 2.4. Electroencephalogram Recording

Electroencephalograms recorded electrical activity at different locations on the scalp, using carefully positioned electrodes [25]. This enables cerebrocortical function in response to noxious stimulation to be identified [25]. Electroencephalogram activity was recorded with Power Lab Biopotential Recordings systems (AD Instruments, Bella Vista, NSW, Australia) immediately upon arrival at UPM, and 4 and 7 days after arrival. On each occasion, the same 24 animals used for blood sampling were used for the EEG recording. Upon entry into the restraining chute and following blood sampling, the animals were allowed to relax for about 10 s and two hydrogel conductive adhesive sterile disposable pads (Kendall^TM^, Covidien, Mansfield, MA, USA) were placed on the zygomatic process of the frontal bone (the inverting–electrode) and the right mastoid process (the non-inverting + electrode) after shaving these areas and cleaning them with 70% alcohol, as recommended by Gibson et al. [26]. The electroencephalographic signal was sampled at a rate of 1 kHz. The raw EEG was resampled with a low pass filter of 200 Hz into theta frequency (4.1 to 8 Hz), delta frequency (0.1 to 4 Hz), beta frequency (12.1 to 20 Hz), and alpha frequency (8.1 to 12 Hz) as described by Zulkifli et al. [27]. Preceding the EEG analysis, the raw EEG recordings were resampled at 1024 Hz and frequencies in the range of 0.1–30 Hz were obtained to reduce the occurrence of artefacts. After the completion of experiments, the EEG data was analyzed offline with the help of the Chart Spectral Analysis Function of Chart 5.0 TM software (Powerlab^TM^ data acquisition system, Sydney, Australia). This software identified potential interferences from concurrent electrocardiograph signals, which were digitally minimized from the raw EEG recordings. Signals were then processed for consecutive non-overlapping 1 s epochs, yielding 60 epochs/min. The root mean square (RMS) of the alpha, beta, delta and theta waves were determined. Two other parameters that were determined were the median frequency (F50; the frequency below 50% of the total power of the EEG) and total power (ptot; the total area under the power spectrum curve).

### 2.5. Statistical Analysis

The structure of the experiment was based on a complete randomized design (CRD). Statistical analysis was carried out by using the General Linear Models (GLM) methodology of Statistical Analysis System (SAS) package Version 9.4 software (SAS Institute Inc., Cary, NC, USA) [28]. Individual animals served as the experimental unit [29]. Data that were not normally distributed were log- or root-transformed prior to analyses. The analysis of the data was performed with sampling time as the primary effect. Comparison among means was made according to Duncan’s multiple range test wherever detectable time effects were present. Statistical significance is considered at *p* < 0.05.

## 3. Results

After sea transport, ALT, AST, and CK were increased and LDH decreased compared to before sea transport (Table 1). After road transport, AST remained elevated, ALT and LDH returned to pre-sea transport levels and CK continued to increase. ALP was increased compared to pre-sea transport. In the recovery period, AST and ALT increased and then decreased, CK declined, LDH remained high and ALP decreased temporarily before returning to levels observed during and after transport.

Compared with samples taken after sea transport, those taken after the subsequent road transport had increased WBC, neutrophils and N:L ratio, and decreased HB and HCT (Table 2). After 4 and 7 days recovery, RBC, HGB and HCT increased—the last two to levels observed after sea transport, and RBC to even higher levels. WBC initially decreased and then increased. PCV, LYM and PLT were not affected by the transport.

**Figure 1:**
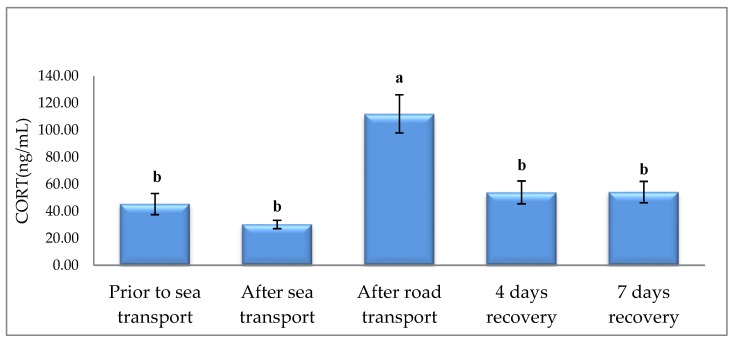
Effects of sea and road transport on serum cortisol concentrations in cattle. The number of animals used in each sampling point was 24 heifers. Means with different letters are significantly different at *p* < 0.05.

**Figure 2:**
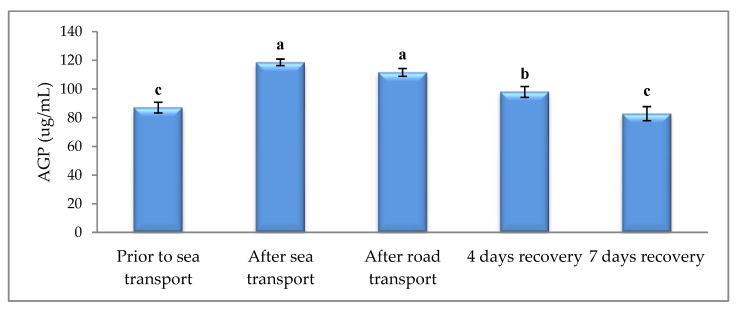
Effects of sea and road transport on serum α-1 glycoprotein (AGP) levels in cattle. The number of animals used in each sampling point was 24 heifers. Means with different letters are significantly different at *p* < 0.05.

**Figure 3:**
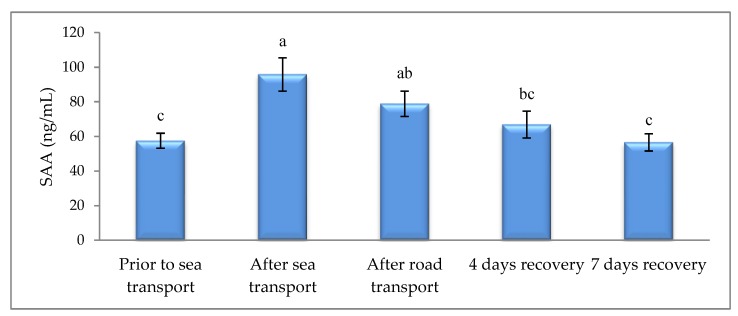
Effects of sea and road transport on serum amyloid-A (SAA) levels in cattle. The number of animals used in each sampling point was 24 heifers. Means with different letters are significantly different at *p* < 0.05.

CORT was increased after road transport, when compared to other sampling times (Figure 1). Sea and road transport resulted in higher AGP (Figure 2) and SAA (Figure 3), compared with prior to sea transport. After 7 d, recovery levels had returned to similar levels observed prior to sea transport.

**Figure 4:**
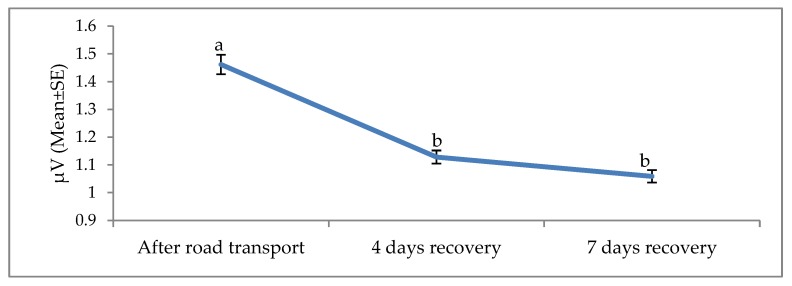
Effects of road transport on the alpha root mean square (RMS) in cattle. The number of animals used in each sampling point was 24 heifers. Means with different letters are significantly different at *p* < 0.05.

**Figure 5:**
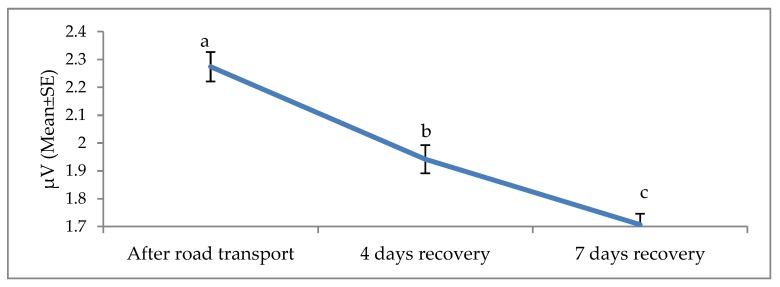
Effects of road transport on the beta root mean square (RMS) in cattle. The number of animals used in each sampling point was 24 heifers. Means with different letters are significantly different at *p* < 0.05.

**Figure 6:**
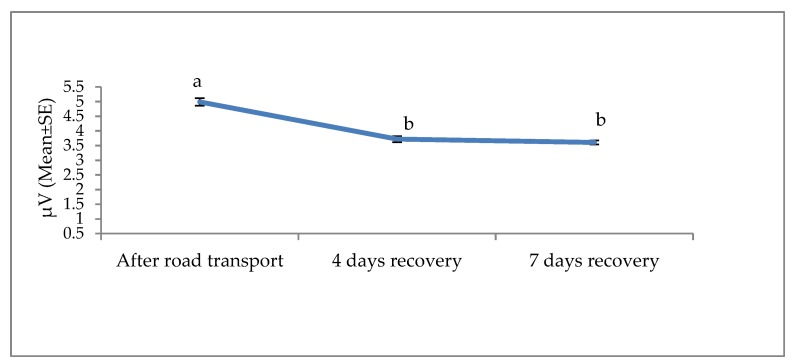
Effects of road transport on the delta root mean square (RMS) in cattle. The number of animals used in each sampling point was 24 heifers. Means with different letters are significantly different at *p* < 0.05.

**Figure 7:**
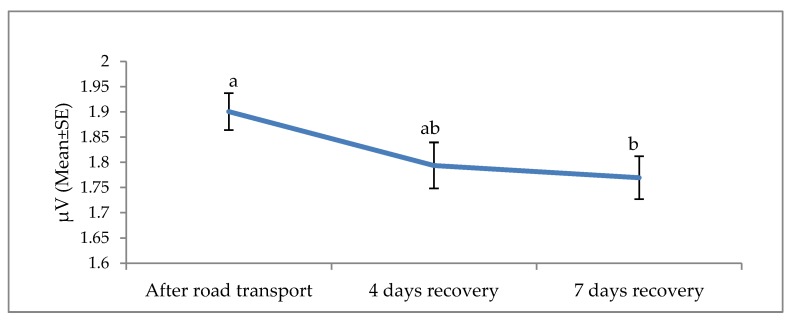
Effects of road transport on the theta root mean square (RMS) in cattle. The number of animals used in each sampling point was 24 heifers. Means with different letters are significantly different at *p* < 0.05.

**Figure 8:**
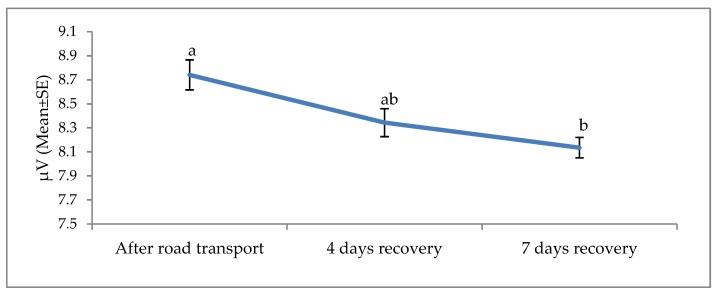
Effects of road transport on total power (ptot) of the EEG in cattle. The number of animals used in each sampling point was 24 heifers. Means with different letters are significantly different at *p* < 0.05.

**Figure 9:**
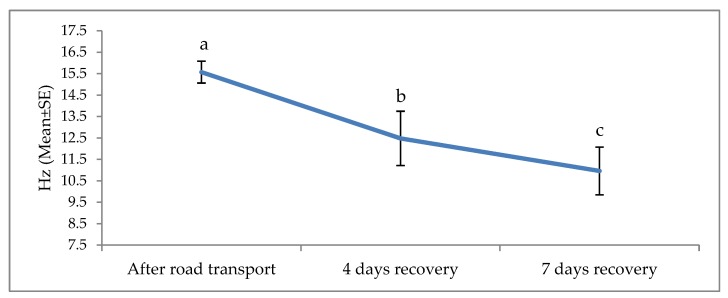
Effects of road transport on the median frequency (MF) of EEG in cattle. The number of animals used in each sampling point was 24 heifers. Means with different letters are significantly different at *p* < 0.05.

The effects of road transport on the alpha, beta, delta and theta waves are illustrated in Figure 4, Figure 5, Figure 6 and Figure 7, respectively. All waves declined from the initial recording after road transport to those recorded after 7 d recovery, and the beta waves showed the most consistent decline. Total power (ptot) also declined over this period (Figure 8) and the median frequency (MF) of the EEG showed a steady decline over the three recordings (Figure 9).

## 4. Discussion

When discussing the CORT results of this experiment, it should be remembered that, because of technical constraints, blood samples were collected at different times of the day and, thus, there is a possibility that the diurnal secretion of the hormone [30] may have affected its concentration. Early studies of the responses of cattle to transportation focused on road transport. Thus, there has been a paucity of research on the response of cattle to both sea and land transport. However, some generalized responses to stress in livestock are well recognized and include elevated CORT [31], as a result of increased corticosteroids and an associated reduction in the number of lymphocytes (lymphopenia or lymphocytopenia) and an increase in the number of neutrophils (neutrophilia) [32]. Our observed increase in cortisol and decreased leukocytes in road-transported cattle are in agreement with earlier work [33,34,35,36]. In the present study, although the animals have been subjected to pre-sea handling, the noted values before sea transport were similar to those at 4 and 7 days recovery. Thus, it appears that the pre-sea CORT concentrations may be considered as control values. The lack of CORT response after sea transport does not preclude the possibility of a prior elevation in circulating levels of CORT, which was eliminated at some stage in the duration of the voyage, especially since the CORT reaction to stress is transient or short lived [31]. Buckam Sporer et al. [37] transported bulls by road for 14 h and recorded a dramatic elevation in CORT following 4.5 h of transit, with the levels diminishing about 4 h post-transport. Our samples of blood were harvested about 12 h after arrival of the vessel at the port. The similar CORT prior to and after sea transport suggests that the animals were either not stressed by the journey or that they recovered from the stress in the vessel before the blood sampling. A similar elevation of cortisol after road but not sea transport has been reported following road transport within Ireland and sea transport to continental Europe [38]. Similarly, elevation in CORT after road transport is confirmed by earlier studies in cattle [39]. It can be speculated that, of the various stressors postulated to affect livestock in road and sea transport, one of the biggest differences is in the motion types experienced. Livestock in road transport experience considerable lateral forces, which may suddenly shunt them sideways when the vehicle rounds a bend. Such shunting forces exist on ships, when “slammers” hit the side of the ship, but this is rare. When tested under simulated ship conditions, livestock are stressed by the major motions experienced, particularly heave [40], with no evidence of a reduction over time, but the duration of testing was short, only one hour. It is conceivable that the motion stress of road transport is greater than that on ships, but much would depend on journey quality and, on ships at least, the positioning of animals. An animal distant from the metacentre of the ship will experience much greater motion.

As part of the acute phase response (APR), the serum proteins that are mainly synthesized by hepatocytes (acute phase proteins, APP) are released as part of the early defense mechanism. This is induced by a range of challenges, such as tissue injury, endotoxin exposure, bacterial infection and inflammation [41,42], to promote healing and re-establish homeostasis. However, another major cause of APR stimulation in cattle is stress [43,44,45,46,47,48,49]. The concentration of APP in blood of calves is increased when they are exposed to abrupt stressors such as weaning [47], transportation [50], housing on slippery floors [43] and mixing of animals [46]. Our study found that SAA and AGP reactions were elicited by both sea and road transport. Similarly, elevated serum levels of SAA and haptoglobin (Hp) have been recorded 48 h following road transport in ewes [51,52] and beef cattle [52]. Similarly, a single session of inescapable tail shock elevated serum AGP and Hp 24 h later in rats [44]. Thus, it can be concluded that both sea and road transport were stressful to the cattle.

Packed cell volumes [53,54], HCT and RBC [55,56] values are considered indicators of dehydration in cattle. High values of PCV can also imply a release of erythrocytes from the spleen to the blood stream as a result of sympathetic-adrenal stimulation [57]. The absence of the effects of road transport on PCV has been observed previously [58]. Despite the availability of drinking water in our research facilities, RBC and HCT values 4 and 7 days post-road transport were higher than those attained immediately after road transport. This suggests a degree of dehydration [55,56]. Increase in HCT values after road transport in cattle has been reported previously [55,59,60].

In the present study, because of technical constraints, we were unable to determine haematological parameters before sea transport. Thus, the baseline values were based on Jackson and Cockroft [61]. According to Jackson and Cockroft [61], all the haematological values presented in Table 2 are within the normal range. However, the elevated WBC, NEU and N:L ratio values after road transport when compared after sea transport suggest stress, instigated by the endogenous release of epinephrine or cortisol. The occurrence of acute leucocytosis is generally distinguished by an increase in circulating neutrophils, and the same observation has also been made in other transportation studies [58,62,63]. The elevated WBC, NEU and N:L ratio following road transport compared to sea transport does not preclude the possibility of a prior elevation in those parameters following the latter. Work by Pettiford et al. [64] in cattle showed elevation in WBC and NEU following 6 h of road transport and the counts started to decline 6 h post-transport. In the present study, blood sampling for haematology was done 12 h after seat transport and, thus, the counts of WBC and NEU may have declined by then. In biomedical studies of mammals, the mechanisms underlying the impact of stress hormones on leukocyte profiles have been investigated [65,66]. As a result of transportation, the neutrophils in circulation were probably young cells from the marginal pool, while the comparative percentage of aged neutrophils reduced. The recorded lower WBC and NEU counts following sea transport when compared to road transport provide further evidence of the stressfulness of the former. However, the magnitude of stress experienced following road transport could be associated with the carry-over effect of sea transport. Although our blood samples were collected, necessarily, 12 and 0 h after arrival, and concur with research by Yagi et al. [33], who road transported dairy cows and found a decrease in neutrophils counts 2 h after unloading. The present findings, as measured by WBC, NEU and N:L ratios, suggest the animals have recovered at 4 or 7 days. It is interesting to note that the HCT values during the recovery period were higher than those after road transport. Animals were provided sufficient drinking space during the recovery period and, thus, it is unlikely that the elevated HCT values are associated with dehydration. There are various factors other than dehydration that may influence HCT values, namely, hemoparasitic infestation, herd management and the season of the year [67,68].

Elevated amounts of AST, ALT and CK serum activities suggest cell muscle damage and muscle fatigue [69]. It has previously been observed that there was an increase in the activities of AST, ALT, and CK after rabbits had been road transported for 126 km [11]. The elevated ALT and AST were suggested to reflect damage to internal organs, hepatopathy, and muscular dystrophy. In our study, ALT and AST levels were still elevated even after 7 days post-road transport, compared to pre-transport. In contrast to this, CK activity diminished after 4 days post-road transport. As observed in several studies [38,70,71], there is a direct positive relation between plasma CK in cattle and the duration of transport. During extraneous activity, CK, which is a highly sensitive enzyme, catalyses the translation of creatine to phosphocreatine to act as an energy reservoir in the tissues. Hence, an increased level of CK activity can be used as an index for assessing muscle fatigue and damage, in particular cardiac muscle and skeletal damage. Increase in the blood activity of CK has a probable relationship with changes in cell membrane permeability as a result of variation in tissue temperature [72]. According to Kannan et al. [73], robust physical activity in goats, such as loading, unloading and herding, were more significant than transportation in assessing the plasma CK activity. The CK values declined significantly at 4 day of recovery. Work by Petifford et al. [64] showed that road transportation for 6 h significantly increased CK levels in yearling cattle and the concentration returned to pre-transport levels after 17 h of recovery.

The electroencephalogram (EEG) records the electrical activity of the cerebral cortical neurons, believed to play a role in pain perception [74]. Activity is measured in four bands, low-frequency delta and theta and high-frequency alpha and beta. EEG responses to pain typically involve a decrease in total power, an increase in 95% spectral edge (the 95th percentile of the power spectrum), and an increase in median frequency (the statistical median of the power spectrum) [25]. This desynchronization has been associated with increased arousal [25]. EEG responses and cortisol concentrations in cattle during the castration process are associated [75], suggesting that EEG may be useful in monitoring pain [75]. Higher beta power in response to stress has been reported in humans, with a positive correlation between beta activity and cortisol levels [76]. To the best of our knowledge, this is the first documented work on EEG and non-pain stimulus in cattle. Work in human beings showed EEG responses to non-painful warm and cold stimuli [22]. Furthermore, work by Tops et al. [23] and McAllister-Williams et al. [24] demonstrated a relationship between exogenous cortisol administration and EEG response in human beings. Hence, it appears non-painful stressful stimuli may induce circulating levels of cortisol and, subsequently, elicit EEG reaction in humans. The present EEG results suggest that road transportation was associated with noxious stimuli. The noted progressive decline in the amplitude of the alpha, beta, delta and theta waves 4 and 7 days post-transport shows a characteristic desynchronization or arousal of the EEG in response to stress associated stimuli. The reduction of the total power, known as desynchronization, was evident in our experiment and evidence of a reduction in cerebral arousal [27,77,78]. It can be concluded that stress attributed to road transport may elicit an EEG reaction and animals recovered at day 4 and 7.

## 5. Conclusions

Sea transport of cattle from Darwin, Australia, to Johore, Malaysia, sufficiently affected the concentration of various physiological and biochemical variables. To conclude, stress was induced, particularly noted in the elevation of acute phase proteins, even though blood samples were taken 12 h after arrival. The lack of circulating cortisol in reaction to sea transport could be attributed to the transient responses of the hormone [31]. The physiological, biochemical and haematological measurements, and EEG data clearly showed that subsequent road transport for 330 km was stressful. There was an indication, as measured by alanine aminotransferase (ALT) and aspartate aminotransferase (AST) that the animals did not fully recover from sea and road transport even by 7 days post-road transport. The prolonged elevation of the levels of AST and ALT suggest hepatopathy, muscular dystrophy and damage to internal organs. However, other stress indicators and EEG data suggest that the animals recovered from the stressfulness of sea and road transport after 4 days. We conclude that both sea and road transport in this journey from Australia to Malaysia are associated with physiological stress but, as measured by hormonal, acute phase proteins, and some haematological and EEG reactions, animals are able to recover from the adverse effects of transportation.

## Figures and Tables

**Table 1:** Effects of sea and road transport on blood biochemical parameters in cattle (Mean ± SE).

Parameter	Prior to Sea Transport	After Sea Transport	After Road Transport	After 4 d Recovery	After 7 d Recovery	*p*-Value
ALT(U/L)	35.00 ^c^ ± 2.47	40.29 ^b^ ± 1.34	28.65 ^c^ ± 1.11	48.90 ^a^ ± 2.09	42.06 ^b^ ± 1.57	<0.0001
ALP(U/L)	138.37 ^b^ ± 2.25	173.30 ^ab^ ± 11.29	175.70 ^a^ ± 11.24	139.65 ^b^ ± 10.89	155.62b ^a^ ± 13.31	0.0219
AST(U/L)	42.00 ^d^ ± 1.10	110.98 ^bc^ ± 3.23	103.36 ^c^ ± 2.92	159.78 ^a^ ± 4.94	119.18 ^b^ ± 6.41	<0.0001
CK(U/L)	155.00 ^c^ ± 5.94	200.45 ^b^ ± 22.96	257.40 ^a^ ± 4.68	160.05 ^c^ ± 3.75	162.31 ^c^ ± 10.03	<0.0001
LDH(U/L)	2137.30 ^a^ ± 81.60	1914.10 ^b^ ± 76.67	2151.35 ^a^ ± 20.42	2272.55 ^a^ ± 98.14	2210.31 ^a^ ± 83.18	0.0150

^a–d^ Means with in the same row with different letters are significantly different at *p* < 0.05. ALT: Alanine aminotransferase, ALP: Alkaline phosphatase, AST: Aspartate aminotransferase, CK: Creatine kinase, LDH: lactate dehydrogenase. The number of animals used in each sampling point was 24 heifers.

**Table 2:** Effects of sea and road transport on haematological parameters in cattle (Mean ± SE).

Parameter	After Sea Transport	After Road Transport	4 Days Recovery	7 Days Recovery	*p*-Value
WBC (×10^9^ /L)	6.09 ^c^ ± 0.37	11.06 ^a^ ± 0.51	6.58 ^c^ ± 0.27	9.08 ^b^ ± 0.44	<0.0001
PCV (%)	39.90 ± 0.70	40.58 ± 0.80	40.25 ± 0.66	39.20 ± 0.50	0.5212
RBC (×10^12^ /L)	9.53 ^bc^ ± 0.32	9.24 ^c^ ± 0.12	10.11b ^a^ ± 0.20	10.26 ^a^ ± 0.22	<0.0001
LYM (×10^9^ /L)	5.52 ± 0.24	5.08 ± 0.28	5.69 ± 0.21	5.44 ± 0.23	0.3391
HGB (g/L)	135.55 ^a^ ± 2.56	124.75 ^b^ ± 1.17	137.7 ^a^ ± 2.35	137.85 ^a^ ± 2.90	<0.0001
HCT (L/L)	0.38 ^a^ ± 0.05	0.35 ^b^ ± 0.04	0.39 ^a^ ± 0.06	0.38 ^a^ ± 0.08	<0.0001
PLT (×10^9^ /L)	333.50 ^a^ ± 22.15	308.87 ^a^ ± 35.25	357.00 ^a^ ± 26.95	371.85 ^a^ ± 29.71	0.4400
NEU (×10^9^ /L)	3.11 ^b^ ± 0.16	4.15 ^a^ ± 0.34	3.24 ^b^ ± 0.14	3.38 ^b^ ± 0.17	<0.0001
N:L ratio	0.56 ^b^ ± 0.07	0.82 ^a^ ± 0.12	0.57 ^b^ ± 0.05	0.62 ^b^ ± 0.07	0.0141

^a–d^ Means in the same row with different letters are significantly different at *p* < 0.05. WBC: White blood cells, PCV: Packed cell volume, RBC: Red blood cells, LYM: Lymphocytes, HGB: Haemoglobin, HCT: Haematocrit, PLT: Platelets, NEU: Neutrophil, N:L ratio: Neutrophil:lymphocyte ratio. The number of animals used in each sampling point was 24 heifers.
